# The promise of RNA-based therapeutics in revolutionizing heart failure management – a narrative review of current evidence

**DOI:** 10.1097/MS9.0000000000001118

**Published:** 2023-07-31

**Authors:** Nicholas Aderinto, Muili O. Abdulbasit, Gbolahan Olatunji, Mariam Edun, Gbolahan Aboderin

**Affiliations:** a Department of Medicine and Surgery, Ladoke Akintola University of Technology, Ogbomoso, Oyo State; b Department of Medicine and Surgery, University of Ilorin, Ilorin, Kwara State, Nigeria

**Keywords:** heart failure, patient outcomes, RNA-based therapeutics

## Abstract

This review elucidates the potential of RNA-based therapeutics to revolutionize heart failure (HF) management. Through a comprehensive analysis of relevant studies, this review reveals the promising prospects of these novel interventions in personalized treatment strategies, targeted modulation of specific molecular pathways, and the attainment of synergistic effects via combination therapies. Moreover, the regenerative capacity of RNA-based therapeutics for cardiac repair and the inherent advantages associated with noninvasive routes of administration are explored. Additionally, the studies accentuate the significance of diligent monitoring of disease progression and treatment response, ensuring safety and considering long-term outcomes. While ongoing research endeavours and technological advancements persist in addressing extant challenges and limitations, the transformative potential of RNA-based therapeutics in HF management offers a beacon of hope for enhanced patient outcomes.

## Introduction

Highlights

- RNA-based therapeutics have a transformative potential in heart failure (HF) management, offering personalized treatment approaches tailored to each patient’s genetic profile and disease characteristics.
- There is the possibility of targeted modulation of specific molecular pathways implicated in HF using RNA-based therapeutics.
- There is the possibility of achieving synergistic effects through the combination of multiple RNA-based therapeutics.

Traditionally, heart failure (HF) has been defined as a condition characterized by the diminished ability of the heart to pump blood or adequately fill it effectively^1^. Alternatively, it can be defined as an abnormality in the structure or function of the heart, resulting in either an insufficient or compensatory cardiac output^2^. However, in 2021, global scientific organizations agreed on a universal definition and classification of HF. According to this consensus, HF is a clinical syndrome that manifests symptoms and/or signs caused by a cardiac abnormality in structure and/or function^3^. This definition is further supported by increased levels of natriuretic peptides and/or objective evidence of congestion in the pulmonary or systemic systems^3^.

HF is a global pandemic that profoundly impacts a staggering number of individuals, with an estimated 26 million people affected worldwide^4^. Moreover, the prevalence of HF continues to rise unabated, posing a substantial and escalating challenge to healthcare systems across the globe^4^. This escalating burden is also reflected in the economy, with the projected costs associated with HF expected to reach alarming levels as the population ages. Notably, in 2012, the overall cost of managing HF in the United States amounted to approximately $30.7 billion^5^. The projected 127% increase in costs is even more concerning, predicted to surge to a staggering $69.8 billion by 2030^5^. Thus, the anticipated financial impact amounts to an estimated cost of approximately $244 per adult in the United States^5^. In light of these figures, it becomes evident that addressing the multifaceted challenges posed by HF is of paramount importance in both the healthcare and economic spheres.

Despite notable progress in therapeutic and preventive approaches, the mortality and morbidity rates associated with HF remain distressingly high, significantly impacting patients’ quality of life. Large, international cohort studies investigating the prognosis of patients with chronic HF, whether managed in the community or outpatient setting, have yielded concerning findings. These studies reveal a 1-year survival rate of ~80–90% for HF patients, in stark contrast to the 97% survival rate observed in the general population^6,7^. Similarly, at the 5-year mark, survival rates in HF patients plummet to around 50–60%, while the general population maintains an 85% survival rate^8^. These disparities underscore the magnitude of the challenge posed by HF and the urgent need for novel and effective interventions to improve outcomes and bridge the gap in survival rates between HF patients and the general population.

In recent years, RNA-based therapeutics have emerged as a promising field with the potential to revolutionize the management of HF. RNA-based therapeutics encompass a range of approaches, including small interfering RNA (siRNA), antisense oligonucleotides (ASOs), and messenger RNA (mRNA) technologies^9^. These innovative therapies offer distinct advantages by targeting specific genetic and molecular pathways involved in the pathogenesis of HF^9^. However, the development of RNA-based therapeutics for HF faces challenges, including delivery methods, stability of RNA molecules, and off-target effects^10^. This study aims to examine the potential of RNA-based therapeutics in revolutionizing the management of HF. It aims to review and summarize the existing evidence surrounding RNA-based therapeutics in the context of HF management.

## Methodology

This review employed a narrative approach to comprehensively analyse and present the current evidence on RNA-based therapeutics in managing HF. The narrative review method allows for a cohesive synthesis of available literature, including preclinical and clinical studies, to comprehensively understand the topic. See Table 1.

**Table 1:** Methodology for literature search

Stage	Description
Total studies obtained	67
Review approach	Narrative approach was employed to comprehensively analyse and present the current evidence on RNA-based therapeutics in managing heart failure (HF). The narrative review method allowed for a cohesive synthesis of available literature, including preclinical and clinical studies, to comprehensively understand the topic.
Database selection	Electronic databases, including PubMed, Embase, Scopus, and Google Scholar, were searched until May 2023 to identify relevant studies.
Search strategy	The search strategy utilized relevant keywords such as ‘RNA-based therapeutics’, ‘heart failure’, ‘gene therapy’, ‘oligonucleotides’, and other related terms to identify potential articles. Additionally, the reference lists of selected articles were manually reviewed to identify additional relevant studies.
Total studies reviewed	20
Inclusion criteria	Studies were included if they met the following criteria: (1) focused on RNA-based therapeutics in the context of HF management, (2) reported findings from preclinical or clinical studies, (3) published in peer-reviewed journals, (4) published between 2000 and May 2023, and (5) written in English.
Exclusion criteria	Editorials, conference abstracts, and studies irrelevant to the topic of RNA-based therapeutics in HF management were excluded from the review.
Data synthesis	The findings from the included studies were synthesized and presented in a narrative format, highlighting significant observations, key findings, and identifying gaps in the current evidence.

A search was conducted in electronic databases, including PubMed, Embase, and Google Scholar, until May 2023. The search strategy employed relevant keywords such as ‘RNA-based therapeutics’, ‘heart failure’, ‘gene therapy’, ‘oligonucleotides’, and other related terms. Additionally, the reference lists of selected articles were manually reviewed to identify additional relevant studies.

Studies were included in this review if they met the following criteria: (1) focused on RNA-based therapeutics in the context of HF management, (2) reported findings from preclinical or clinical studies, (3) published in peer-reviewed journals, (4) published between 2000 and May 2023, and (5) written in English. Editorials, conference abstracts, and studies irrelevant to the topic of interest were excluded. The findings from the included studies were synthesized and presented in a narrative format, highlighting significant observations and key findings and identifying gaps in the current evidence.

## Mechanisms of RNA-based therapeutics in HF

**Figure 1:**
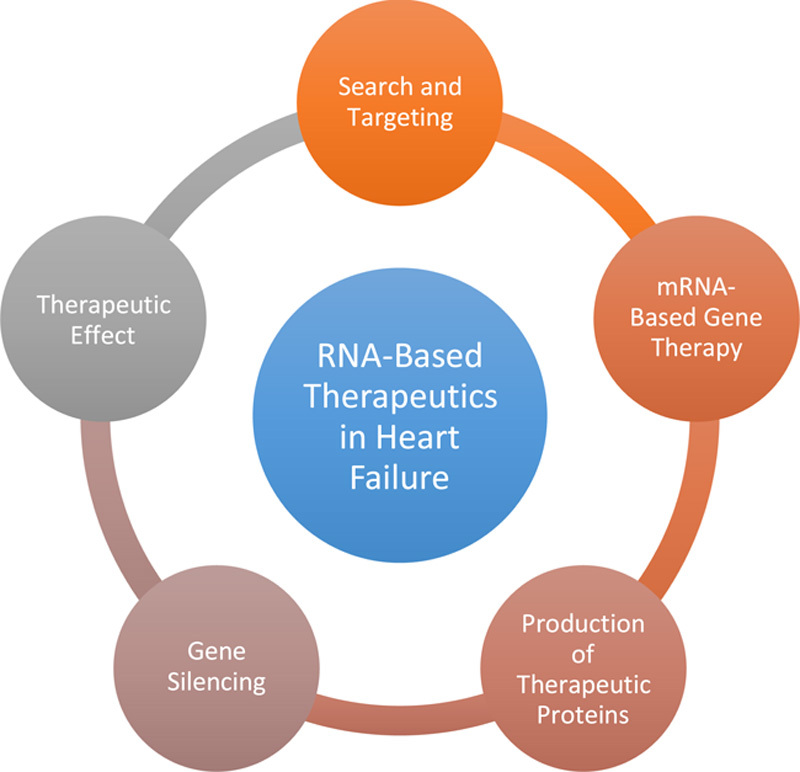
Mechanisms of RNA-based therapeutics in heart failure management.

RNA-based therapeutics have emerged as a promising approach for treating various diseases, including HF^9^. See Figure 1. These therapeutics utilize different types of RNA molecules, such as ASOs, siRNAs, and mRNAs, to modulate gene expression and restore proper cellular function^10^.

### Antisense oligonucleotides

ASOs have garnered significant attention as a promising class of therapeutic molecules in molecular medicine^11^. These short, single-stranded RNA molecules are meticulously designed to selectively bind to specific mRNA molecules, exerting a regulatory effect on gene expression^12^. ASOs have demonstrated remarkable potential in targeting disease-causing genes or aberrant gene products, positioning them as a valuable tool for addressing various genetic disorders, including HF^13^.

ASOs offer a highly targeted approach to modulating gene expression and potentially rectifying the underlying molecular defects associated with HF^13^. Their mechanism of action hinges upon the ability to engage in Watson–Crick base pairing with the complementary mRNA sequence^14^. The binding between ASOs and mRNA can occur at different regions of the mRNA molecule, depending on the desired therapeutic outcome^15^. ASOs can be engineered to bind to the coding region of mRNA, resulting in the degradation of the targeted mRNA molecule through cellular ribonucleases^16^. This degradation process effectively impedes mRNA translation into protein, thereby reducing the expression of the disease-causing gene product^16^. Alternatively, ASOs can target the non-coding regions of mRNA, such as the 5ʹ untranslated region (UTR) or the 3ʹ UTR^17^. By engaging with these regions, ASOs can disrupt post-transcriptional processes, including mRNA splicing, stability, or transport^17^. Through modulation of these processes, ASOs can influence the levels of functional mRNA available for translation, ultimately impacting protein production^17^.

The design and development of ASOs for HF therapy necessitate careful consideration of several critical factors. The selection of target genes to be modulated assumes paramount importance^18^. This entails identifying genes that play a substantial role in the development or progression of HF and are amenable to ASO-mediated regulation. Advances in genomic research and evolving understanding of the molecular mechanisms underpinning HF have yielded valuable insights into potential gene targets for ASO therapy^19^. Once the target gene is identified, the ASO sequence is meticulously crafted to complement the specific mRNA region of interest. Multiple modifications can be introduced to enhance the stability, specificity, and delivery efficiency of ASOs. Chemical modifications, such as phosphorothioate linkages, can bolster ASO stability by shielding them from cellular nucleases responsible for degradation^20^. Additionally, conjugation with ligands or nanoparticles can facilitate the delivery of ASOs to the desired tissue or cells, enhancing their therapeutic efficacy in treating HF.

### Small interfering RNAs

siRNAs, or small interfering RNAs, represent a pivotal class of RNA molecules that exert significant control over gene expression through RNA interference (RNAi)^21^. Typically consisting of double-stranded RNA molecules ~20–25 nucleotides long, siRNAs can specifically target and degrade complementary mRNA molecules, thereby serving as a potent tool for gene silencing and therapeutic intervention^22^. In managing HF, siRNAs hold substantial promise as a potential treatment strategy by selectively silencing genes implicated in the disease’s pathogenesis. By offering a targeted approach to gene silencing, siRNAs hold the potential to mitigate disease symptoms and enhance cardiac function.

The mechanism of action underlying siRNAs centres on their incorporation into the RNA-induced silencing complex (RISC)^23^. Within RISC, the siRNA duplex undergoes unwinding, forming an active RISC complex with a single-stranded guide RNA^23^. This guide RNA, also called the antisense strand, exhibits complementarity to the target mRNA sequence^23^. The RISC complex then binds to the target mRNA through base pairing between the guide RNA and the mRNA, ultimately leading to the cleavage and subsequent degradation of the mRNA molecule. This degradation effectively halts the translation of the mRNA into protein, thereby diminishing the expression of the target gene.

The design and development of siRNAs for HF therapy demand meticulous consideration to ensure their specificity, stability, and effective delivery to the intended target tissue or cells. An integral initial step involves carefully selecting the target gene or genes, necessitating a comprehensive understanding of the molecular pathways and genetic elements implicated in HF^24^. Following the identification of the target gene, the siRNA sequence is thoughtfully designed to exhibit complementarity to the specific mRNA region of interest–the design process endeavours to ensure the siRNA’s specificity while minimizing the potential for off-target effects^24^. Multiple algorithms and software tools have been developed to aid in predicting efficient siRNA sequences based on considerations such as target accessibility, secondary structure, and the avoidance of potential off-target sites^25^. By adhering to these design principles, researchers strive to optimize the therapeutic potential of siRNAs for managing HF.

### Messenger RNA therapies

mRNA-based therapies have emerged as a highly promising modality within molecular medicine^26^. These innovative therapeutic approaches involve the introduction of exogenous mRNA molecules into target cells, facilitating the translation of the mRNA into therapeutic proteins^26^. In the specific context of HF, mRNA therapies hold immense potential for addressing the underlying molecular defects, restoring normal cardiac function, and modulating critical signalling pathways implicated in disease pathogenesis^27^. The notable triumph of mRNA vaccines against COVID-19 has underscored mRNA’s remarkable versatility and efficacy as a therapeutic tool, further bolstering the prospects of mRNA-based therapies in HF management^28^.

Fundamentally, mRNA-based therapies involve the delivery of exogenous mRNA molecules encoding the desired therapeutic protein into target cells^26^. Once inside the cellular milieu, these mRNA molecules are readily recognized by the cellular machinery responsible for protein synthesis, namely the ribosomes. Consequently, the ribosomes translate mRNA, faithfully converting the mRNA sequence into the corresponding protein. Through this mechanism, mRNA therapy enables the expression of the therapeutic protein within the target cells.

One crucial application of mRNA-based therapy in the HF domain is restoring missing or defective proteins^29^. In certain instances of HF, the absence or malfunction of specific proteins may stem from genetic mutations or other aetiological factors^29^. By delivering exogenous mRNA that encodes the missing or defective protein, mRNA therapy imparts the instructions for the target cells to synthesize and produce the functional protein, thus potentially rectifying cellular deficiencies, restoring normal physiological functions, and enhancing overall cardiac performance^30^. Additionally, mRNA-based therapy exhibits promise in the modulation of signalling pathways intricately associated with HF^31^. The pathogenesis of HF entails the dysregulation of multiple signalling cascades that contribute to underlying cardiac dysfunction^32^. By delivering exogenous mRNA molecules that encode specific signalling molecules or regulators, mRNA therapy can effectively modulate these aberrant pathways, reinstating their delicate balance and potentially alleviating disease symptoms.

Moreover, the versatility of mRNA-based therapies extends to the capacity for eliciting specific cellular responses or manipulating gene expression patterns^33^. By meticulously engineering the mRNA sequence or incorporating regulatory elements, researchers can precisely regulate the translation of the mRNA into protein or influence various cellular processes. This capability allows for fine-tuning gene expression patterns and modulating cellular functions intricately associated with HF.

## Preclinical studies on RNA-based therapeutics in HF

Preclinical studies exploring the application of RNA-based therapeutics in the context of HF have yielded invaluable insights, shedding light on the prospective use of RNA molecules as therapeutic agents for treating this intricate cardiovascular disorder. See Table 2. These studies, conducted primarily in animal models and in-vitro systems, have concentrated on harnessing the distinctive characteristics of RNA to finely regulate gene expression, modulate protein function, and influence key cellular processes intricately involved in the pathophysiology of HF.

**Table 2:** Summary of preclinical studies on RNA-based therapeutics in heart failure

References	Approach	Key findings
Dickinson *et al*.^34^	Therapeutic potential of miRNAs	Identified miR-19b as a potential biomarker for heart failure treatment response.
		Highlighted the significant increase in plasma levels of miR-19b following anti-miR-208a treatment.
		Suggested miR-19b as a potential biomarker for assessing the effectiveness of therapeutic interventions.
Zhang *et al*.^35^	Therapeutic potential of miRNAs	Demonstrated the protective effects of miR-125b in heart failure.
		Showed improvements in cardiac function, reduced fibrosis and damage, and decreased levels of specific markers.
		Proposed miR-125b as a potential therapeutic target for heart failure.
Hinkel *et al*.^36^	Anti-miR-21 therapy	Significant reduction in fibrosis, improved cardiac function, and increased survival rates in heart failure mice.
		Highlighted the therapeutic potential of anti-miR-21 therapy for mitigating cardiac fibrosis and remodelling.
Suckau *et al*.^37^	RNA interference (RNAi)	Improved calcium handling, enhanced contractile function, and reduced adverse remodelling in heart failure mice.
		Demonstrated the potential of RNAi-mediated downregulation of phospholamban for restoring calcium homeostasis.
Li *et al*.^38^	RNA aptamer to disrupt OPN signalling	Attenuated inflammation, reduced fibrosis, and improved cardiac function in heart failure.
		Highlighted the potential of RNA aptamers as targeted therapeutics to disrupt specific signalling pathways.
Thum *et al*.^39^	Role of miR-21 in cardiac fibrosis	MiR-21 promotes fibroblast activation and collagen synthesis in cardiac fibrosis.
	and remodelling	Inhibiting miR-21 expression attenuated fibroblast activation, reduced collagen deposition, and improved function.
Sassi *et al*.^40^	Role of miR-29 in hypertrophy and fibrosis	MiR-29 downregulation correlates with increased Wnt signalling in cardiac hypertrophy and fibrosis.
		Modulating miR-29 levels reduced hypertrophic and fibrotic responses.
Methawasin *et al*.^41^	Titin compliance modulation (HFpEF)	Modulating RBM20 expression increased compliant titin isoforms, improving diastolic function in HFpEF.
Radke *et al*.^42^	ASOs targeting RBM20	Investigated ASOs targeting RBM20 as a potential therapeutic approach for HFpEF.
		Aimed to modulate titin compliance and improve diastolic function in HFpEF patients.
Beverborg *et al*.^43^	ASOs targeting phospholamban	Explored the use of ASOs targeting phospholamban as a potential therapeutic option for heart failure.
Morihara *et al*.^44^	ASOs targeting phospholamban	Inhibited phospholamban expression, resulting in improved calcium handling and cardiac function in HF.

### Therapeutic potential of microRNAs (miRNAs) in HF

The therapeutic potential of miRNAs in HF has been explored in two notable studies by Dickinson *et al*. and Zhang *et al*., shedding light on the significance of specific miRNAs in this complex cardiovascular condition^34,35^. Dickinson *et al*.’s study focused on the role of plasma miRNAs as potential biomarkers for HF treatment success and disease progression. Their findings revealed distinct variations in miRNA expression among different treatment groups, particularly highlighting the significant increase in plasma levels of miR-19b following anti-miR-208a treatment. This observation suggests that miR-19b holds promise as a potential biomarker for assessing the effectiveness of therapeutic interventions in HF. This study opens up possibilities for personalized medicine and targeted interventions in HF management by identifying specific miRNAs associated with treatment response.

In contrast, Zhang *et al.*’s study centred on elucidating the role of miR-125b in HF. Their study utilized a mouse model and demonstrated that miR-125b expression was downregulated in heart tissues of HF mice. However, when miR-125b was overexpressed, it resulted in notable improvements in cardiac function, reduced myocardial fibrosis and damage, and decreased levels of atrial natriuretic peptide (ANP), B-type/brain natriuretic peptide (BNP), and major histocompatibility complex (MHC). These findings indicate that miR-125b exerts protective effects in HF and may serve as a potential therapeutic target. Manipulating miR-125b levels could offer a novel approach to mitigating the detrimental effects of HF and improving cardiac function.

By highlighting the therapeutic potential of specific miRNAs, both studies contribute to understanding the intricate mechanisms underlying HF and present opportunities for developing targeted interventions. The identification of miR-19b as a potential biomarker for assessing treatment effectiveness and the protective effects of miR-125b offer new avenues for personalized and tailored approaches in managing HF. Further exploration and validation of these miRNAs and their associated pathways may pave the way for novel therapeutic strategies to improve outcomes in individuals with HF.

### Promising RNA-based therapeutics for HF

The studies conducted by Hinkel *et al*., Suckau *et al*., and Li *et al*. provide valuable insights into the potential of RNA-based therapeutics for HF. Each study focuses on a different approach, targeting specific molecules or signalling pathways to improve cardiac function and prevent cardiac dysfunction.

Hinkel *et al*. aimed to improve cardiac function by targeting miR-21 using anti-miR-21 therapy^36^. miR-21 has been implicated in cardiac fibrosis and adverse remodelling in HF. In their study, the researchers utilized ASOs to inhibit miR-21. They observed a significant reduction in fibrosis, improved cardiac function, and increased survival rates in a mouse model of HF. This highlights the therapeutic potential of anti-miR-21 therapy as a promising strategy for mitigating the detrimental effects of cardiac fibrosis and remodelling in HF.

Suckau *et al*. focused on RNAi to enhance calcium management and improve cardiac function^37^. They targeted phospholamban (PLN), a protein regulating cardiomyocyte calcium cycling. By using siRNAs to silence PLN expression, they demonstrated improved calcium handling, enhanced contractile function, and reduced adverse remodelling in a mouse model of HF. These findings suggest that RNAi-mediated downregulation of PLN holds therapeutic potential for restoring calcium homeostasis and improving cardiac function in HF.

Li *et al*. explored using an RNA aptamer to disrupt osteopontin (OPN) signalling and prevent cardiac dysfunction^38^. OPN is a protein associated with inflammation and adverse cardiac remodelling. The researchers designed an RNA aptamer that binds to OPN, inhibiting its signalling and subsequent pathological effects. In a rat model of HF, the RNA aptamer successfully attenuated inflammation, reduced fibrosis, and improved cardiac function. This study highlights the potential of RNA aptamers as targeted therapeutics to disrupt specific signalling pathways and mitigate the detrimental effects of cardiac dysfunction in HF.

### Role of miRNAs in cardiac fibrosis and remodelling

The studies conducted by Thum *et al*. and Sassi *et al*. shed light on the role of miRNAs in cardiac fibrosis and remodelling, providing insights into the molecular mechanisms underlying these pathological processes.

Thum *et al*. investigated the involvement of miR-21 in cardiac fibrosis and remodelling, specifically focusing on its impact on cardiac fibroblasts^39^. They demonstrated that miR-21 is upregulated in response to profibrotic stimuli and significantly promotes fibroblast activation and collagen synthesis. By manipulating miR-21 levels *in vitro* and in a mouse model of cardiac fibrosis, the researchers showed that inhibiting miR-21 expression can attenuate fibroblast activation, reduce collagen deposition, and improve cardiac function. These findings highlight the importance of miR-21 in mediating cardiac fibrosis and remodelling, suggesting it is a potential therapeutic target for intervention in fibrotic heart diseases.

Sassi *et al*. focused on the role of miR-29 in hypertrophy and fibrosis of the heart, particularly its control of Wnt signalling^40^. The researchers demonstrated that miR-29 is downregulated in cardiac hypertrophy and fibrosis, and its decreased expression correlates with the upregulation of Wnt signalling components. By modulating miR-29 levels *in vitro* and in a mouse model of cardiac hypertrophy, they revealed that miR-29 exerts inhibitory effects on Wnt signalling, leading to reduced hypertrophic and fibrotic responses. This study highlights the regulatory role of miR-29 in cardiac remodelling and fibrosis, specifically through its interaction with Wnt signalling pathways. It suggests that targeting miR-29 or modulating Wnt signalling could have therapeutic implications for managing cardiac hypertrophy and fibrotic remodelling.

These studies provide valuable insights into the involvement of specific miRNAs, such as miR-21 and miR-29, in cardiac fibrosis and remodelling. Understanding the molecular mechanisms and signalling pathways controlled by these miRNAs offers potential targets for therapeutic intervention in fibrotic heart diseases. Further research is needed to explore the broader miRNA landscape and the intricate regulatory networks involved in cardiac fibrosis and remodelling, aiming to develop effective miRNA-based therapies for patients suffering from these cardiac pathologies.

### Titin compliance modulation for HF with preserved ejection fraction (HFpEF)

The study conducted by Methawasin *et al*. investigated the modulation of titin compliance as a potential therapeutic strategy for HFpEF, a condition characterized by diastolic dysfunction^41^.

The researchers focused on targeting the splicing factor RBM20, which plays a crucial role in the alternative splicing of titin, a giant protein responsible for cardiac muscle elasticity. Altered splicing of titin isoforms has been implicated in the development of diastolic dysfunction in HFpEF. By manipulating RBM20 expression in a mouse model of HFpEF, Methawasin *et al*. aimed to improve titin compliance and subsequently enhance diastolic function.

The study demonstrated that reducing RBM20 expression led to an increase in the expression of compliant titin isoforms. This modulation of titin splicing improved diastolic function, characterized by enhanced myocardial relaxation and reduced myocardial stiffness. The findings suggested that targeting RBM20-mediated titin splicing could be a viable therapeutic approach for treating HFpEF by restoring normal titin compliance and improving diastolic function.

By identifying RBM20 as a key player in the regulation of titin splicing and demonstrating its potential as a therapeutic target, Methawasin *et al*. shed light on the importance of titin compliance modulation in the management of HFpEF. This study provides a promising avenue for future research and development of therapies to restore titin elasticity and improve diastolic function in patients with HFpEF. However, further studies, including clinical trials, are needed to validate the effectiveness and safety of this approach in humans before it can be translated into clinical practice.

### ASOs targeting RBM20 and PLN as potential therapeutic strategies

The studies conducted by Radke *et al*., Beverborg *et al*., and Morihara *et al*. focused on the potential therapeutic strategies of using ASOs to target specific molecules involved in cardiac function and HF.

Radke *et al*. investigated the use of ASOs targeting RBM20, a splicing factor involved in cardiac titin isoform expression, as a potential therapeutic approach for HFpEF. By targeting RBM20, the study aimed to modulate titin compliance and improve diastolic function in HFpEF patients^42^. The findings of this study may provide valuable insights into the development of targeted therapies for HFpEF by directly manipulating the splicing of titin isoforms.

Beverborg *et al*. and Morihara *et al*., an unknown author’s study on PLN, explored the use of ASOs targeting PLN as a potential therapeutic option for HF^43,44^. PLN is a regulatory protein involved in calcium handling in cardiac cells, and its dysfunction can contribute to cardiac dysfunction and HF. By utilizing ASOs to target PLN, these studies aimed to modulate its expression and activity, potentially improving calcium management and overall cardiac function.

Using ASOs as a therapeutic strategy offers a targeted approach to modulating gene expression and protein function. ASOs are designed to bind to specific RNA sequences and can either inhibit or enhance gene expression, depending on their mechanism of action. By utilizing ASOs to target RBM20 or PLN, the studies aimed to modify the expression or function of these molecules, potentially restoring normal cardiac function and alleviating HF symptoms.

These studies highlight the potential of ASOs as a therapeutic tool for manipulating specific molecular targets involved in cardiac function and HF. ASOs offer the advantage of high specificity, allowing for precise modulation of gene expression or protein activity. However, further research is needed to evaluate the safety, efficacy, and long-term effects of ASO-based therapies in human subjects. Clinical trials and translational studies will be crucial to determine the potential of ASOs targeting RBM20 and PLN as viable therapeutic strategies for HF.

## Clinical trials and applications of RNA-based therapeutics in HF

Clinical trials and applications of RNA-based therapeutics in HF have provided valuable insights into these innovative approaches’ feasibility, safety, and efficacy in humans. See Table 3. These trials aim to translate the promising preclinical findings into clinically relevant interventions for treating HF, a complex and debilitating cardiovascular condition.

**Table 3:** Clinical trials and applications of RNA-based therapeutics in heart failure

References	Objective and focus	Key findings
Kumarswamy *et al*.^45^	lncRNAs as biomarkers for heart failure diagnosis	Identified lncRNAs, including LIPCAR, as potential diagnostic biomarkers for heart failure.
Zangi *et al*.^46^	lncRNAs as biomarkers for heart failure prognosis	LIPCAR showed increased expression in heart failure patients and was associated with higher risk of cardiovascular deaths.
Yang *et al*.^47^	Role of non-coding RNAs in heart failure pathogenesis	lncRNAs, miRNAs, and mRNAs exhibited differential expression between ischaemic and non-ischaemic heart failure.
Wang *et al*.^48^	Competing endogenous RNA (ceRNA) networks in heart failure	Identified immune and inflammatory pathways enriched in ceRNA network associated with heart failure.
Endo *et al*.^49^	MicroRNAs (miRNAs) as biomarkers for heart failure	miR-210, miR-1, miR-21, miR-23, miR-423-5p, and miR-132 showed altered expression in heart failure patients.
Schneider *et al*.^50^	miRNAs as biomarkers for heart failure	miR-21, miR-126, miR-423-5p, and miR-199a-3p showed potential as prognostic biomarkers for heart failure.
Masson *et al*.^51^	miRNAs as biomarkers for heart failure	miR-122, miR-455-3p, and miR-574-5p were associated with cardiovascular events and mortality in heart failure patients.
Ovchinnikova *et al*.^52^	miRNAs as biomarkers for heart failure	miR-125b, miR-130a, miR-145, and miR-195 showed potential as prognostic biomarkers for heart failure.
Täubel *et al*.^53^	Antisense oligonucleotides (ASOs) as therapeutic approach	ASOs targeting miR-132 demonstrated potential efficacy in reducing NT-proBNP levels, indicating improved cardiac function.
Wang *et al*.^54^	Potential role of exosomal miRNAs in cardiac fibrosis	miR-21, miR-425, and miR-744 were downregulated in heart failure patients’ plasma exosomes, and overexpression attenuated fibrogenesis in cardiac fibroblasts by targeting TGFβ1.

### Long non-coding RNAs (lncRNAs) as biomarkers

The studies by Kumarswamy *et al*. and Zangi *et al*. have shed light on the potential of lncRNAs as biomarkers for HF. These lncRNAs, such as LIPCAR (Long Intergenic Non-Protein Coding RNA Regulator of Cardiac Hypertrophy), have shown distinct expression patterns associated with cardiac remodelling and can serve as diagnostic biomarkers for HF.

In their study, Kumarswamy *et al*. aimed to identify lncRNAs that could be biomarkers for HF diagnosis^45^. They conducted a comprehensive analysis of lncRNA expression profiles in HF patients and compared them to those in healthy individuals. Their analysis identified several lncRNAs, including LIPCAR, that exhibited significant differential expression between the two groups. Furthermore, they found that the expression levels of these lncRNAs correlated with the severity of cardiac remodelling. The study suggested that measuring the levels of these lncRNAs in circulating blood samples could provide a noninvasive means of diagnosing HF.

Similarly, Zangi *et al.* (2013) focused on identifying and validating lncRNAs as potential biomarkers for HF^46^. They conducted a genome-wide analysis of lncRNA expression in HF patients and identified a panel of differentially expressed lncRNAs. Among these lncRNAs, LIPCAR was again found to be highly upregulated in HF patients. Importantly, the study also demonstrated that the increased expression of LIPCAR was associated with an increased risk of future cardiovascular deaths. This suggests that LIPCAR could not only serve as a diagnostic biomarker but also as a prognostic indicator for adverse outcomes in HF patients.

These studies highlight the potential of lncRNAs, particularly LIPCAR, as valuable biomarkers for HF. The distinct expression patterns of these lncRNAs in HF patients provide insights into the underlying molecular mechanisms involved in cardiac remodelling and disease progression. However, further research and validation are necessary to confirm the diagnostic and prognostic utility of lncRNAs in larger patient cohorts. Nonetheless, these findings pave the way for developing noninvasive diagnostic tools and personalized therapies for HF based on lncRNA biomarkers.

### Role of non-coding RNAs in HF pathogenesis

The study by Yang *et al*. investigated the role of non-coding RNAs, including lncRNAs, miRNAs, and mRNAs, in the pathogenesis of HF^47^. By comparing the expression profiles of these non-coding RNAs in failing human hearts, the researchers aimed to understand their involvement in HF and identify potential molecular mechanisms underlying the disease.

The study found that the expression profiles of lncRNAs could distinguish between ischaemic and non-ischaemic HFs. Ischaemic HF is caused by the reduced blood supply to the heart muscle due to coronary artery disease. In contrast, non-ischaemic HF has different underlying causes, such as hypertension, genetic factors, or viral infections. This differential expression of lncRNAs suggests their potential role in contributing to the specific pathogenesis of different types of HF.

Furthermore, the study highlighted the involvement of other non-coding RNAs, such as miRNAs and mRNAs, in HF. These non-coding RNAs regulate gene expression and modulate various cellular processes. The altered expression of miRNAs and mRNAs in failing human hearts suggests their potential contribution to the dysregulation of cardiac function and remodelling processes in HF.

### Competing endogenous RNA (ceRNA) networks in HF

The study by Wang *et al.* (2019) focused on constructing a ceRNA network associated with HF^48^. The ceRNA network was built using differentially expressed lncRNAs, miRNAs, and mRNAs to understand their interactions and potential roles in HF pathogenesis.

The study found that immune and inflammatory pathways enriched the ceRNA network associated with HF. This suggests that the identified lncRNAs, miRNAs, and mRNAs may play a crucial role in modulating immune responses and inflammatory processes in the context of HF. These findings highlight the importance of immune and inflammatory dysregulation in the development and progression of HF.

Within the ceRNA network, the study identified hub nodes, including specific lncRNAs and miRNAs, that exhibited significant interactions with multiple target mRNAs. These hub nodes are considered key regulators within the ceRNA network and have the potential to serve as diagnostic biomarkers and therapeutic targets for HF. By targeting these hub nodes, it may be possible to modulate the ceRNA interactions and restore normal gene expression patterns, thereby potentially improving cardiac function and mitigating HF progression.

Identifying specific lncRNAs, miRNAs, and mRNAs within the ceRNA network associated with HF provides valuable insights into the disease’s molecular mechanisms. Moreover, the enrichment of immune and inflammatory pathways highlights the importance of these processes in HF pathogenesis. The identified hub nodes within the ceRNA network represent promising targets for future diagnostic and therapeutic strategies for HF.

However, further studies are needed to validate the functional significance of the identified lncRNAs, miRNAs, and mRNAs within the ceRNA network and to elucidate their precise roles in immune and inflammatory modulation in HF. Additionally, understanding the dynamic nature of the ceRNA network and its interactions with other regulatory networks will provide a more comprehensive understanding of the complex molecular mechanisms involved in HF.

### miRNAs as biomarkers

The studies by Endo *et al*., Schneider *et al*., Masson *et al*., and Ovchinnikova *et al*. investigated the potential of miRNAs as biomarkers for HF^49–52^. These studies aimed to identify specific miRNAs that exhibit differential expression patterns in HF patients and explore their diagnostic potential in predicting prognosis and risk stratification.

The findings from these studies revealed several miRNAs that were consistently associated with HF. Among the identified miRNAs were miR-210, miR-1, miR-21, miR-23, miR-423-5p, and miR-132. These miRNAs exhibited altered expression levels in HF patients compared to healthy individuals, suggesting their involvement in the pathogenesis of the disease.

The dysregulation of these miRNAs indicates their potential as diagnostic biomarkers for HF. By assessing the expression levels of these miRNAs in patients, clinicians may be able to identify individuals at higher risk for HF or monitor disease progression. These miRNAs can serve as noninvasive markers that provide valuable information about the cardiac status and help guide treatment decisions.

Furthermore, the identified miRNAs also hold promise as prognostic biomarkers. Their expression patterns were found to correlate with clinical outcomes and disease severity. Elevated levels of certain miRNAs, such as miR-21 and miR-132, were associated with worse prognosis, while others, like miR-423-5p, were linked to improved outcomes. By assessing the expression levels of these miRNAs, healthcare professionals can potentially stratify patients based on their risk and tailor treatment plans accordingly.

It is important to note that the identified miRNAs are not standalone biomarkers but should be considered as part of a panel or in conjunction with other clinical parameters. Combining miRNA analysis with traditional diagnostic methods can enhance the accuracy and reliability of HF diagnosis and prognosis assessment.

Although these studies highlight the potential of miRNAs as biomarkers for HF, further research is needed to validate their utility in larger patient populations and diverse cohorts. Additionally, investigating the underlying molecular mechanisms by which these miRNAs contribute to HF and pathogenesis will deepen our understanding of the disease and potentially uncover novel therapeutic targets.

### ASOs as therapeutic approaches

The study by Täubel *et al*. focused on the use of ASOs as a therapeutic approach for HF, specifically targeting miR-132^53^. ASOs are short synthetic strands of nucleotides that can specifically bind to RNA molecules and modulate their function.

The study investigated the safety, tolerability, and potential efficacy of ASOs targeting miR-132 in HF patients. The results showed promising outcomes. The ASO treatment was well-tolerated by the patients, indicating its safety profile. Additionally, the ASO treatment demonstrated potential efficacy in reducing the levels of NT-proBNP, a biomarker associated with HF severity, suggesting an improvement in cardiac function.

Furthermore, the study observed improved cardiac electrical conduction in response to ASO treatment. This finding suggests that targeting miR-132 using ASOs may positively impact the underlying mechanisms contributing to HF, potentially leading to improved cardiac performance and clinical outcomes.

The use of ASOs as therapeutic agents in HF holds great promise. ASOs offer the advantage of targeted therapy by specifically modulating the expression or function of disease-associated genes or non-coding RNAs. By targeting miR-132, which has been implicated in HF pathogenesis, ASOs have the potential to alter the disease processes and mitigate its detrimental effects on the heart.

However, it is important to note that the study by Täubel *et al*. represents an early investigation into the therapeutic potential of ASOs in HF. While the results are promising, further research is needed to validate these findings in larger clinical trials involving a diverse range of patients. Robust clinical trials will clarify the safety, efficacy, and long-term effects of ASO treatment in HF.

### Potential role of exosomal miRNAs in cardiac fibrosis

The study by Wang *et al*. explored the potential role of exosomal miRNAs in cardiac fibrosis, a common pathological process observed in HF^54^. The researchers focused on investigating candidate miRNAs’ expression patterns and functional implications in plasma exosome samples obtained from HF patients.

The study identified several miRNAs that exhibited altered expression in HF patients compared to healthy individuals. Specifically, miR-21, miR-425, and miR-744 were downregulated in HF patients’ plasma exosomes. The observed downregulation of these miRNAs suggested their involvement in developing and progressing cardiac fibrosis.

Furthermore, the study explored the functional implications of these altered miRNAs in cardiac fibrosis. The researchers demonstrated that overexpression of miR-425 and miR-744 in cardiac fibroblasts led to attenuation of fibrogenesis, the process of excessive collagen deposition and fibrotic tissue formation in the heart. The miRNAs achieved this effect by directly targeting and inhibiting the expression of TGFβ1, a key mediator in promoting fibrosis.

The findings of this study shed light on the potential role of exosomal miRNAs, particularly miR-21, miR-425, and miR-744, in modulating cardiac fibrosis in HF. By demonstrating their downregulation in HF patients and their ability to attenuate fibrogenesis in cardiac fibroblasts, these miRNAs present themselves as potential therapeutic targets for combating cardiac fibrosis and its associated complications.

It is important to note that further research is necessary to fully understand the mechanisms underlying the regulation and function of exosomal miRNAs in cardiac fibrosis. Additionally, the clinical relevance and potential therapeutic applications of targeting these miRNAs require validation through additional studies, including in-vivo experiments and clinical trials.

## Challenges and future directions

The development and application of RNA-based therapeutics in the context of HF face several challenges and limitations. These challenges can affect various stages of the therapeutic development process, including target identification, delivery, off-target effects, immunogenicity, and long-term safety. Understanding and addressing these limitations are essential for successfully translating RNA-based therapeutics into clinical practice for HF treatment.

### Limitations faced in the development and application of RNA-based therapeutics

Identifying and selecting appropriate target genes for RNA-based therapeutics can be challenging. HF is a complex condition with multiple molecular pathways involved^12^. Identifying the most relevant target genes and ensuring their specificity is crucial to avoid off-target effects and unintended consequences. Advances in genomics, transcriptomics, and systems biology are aiding in identifying suitable targets; however, further research is needed to validate and refine these targets. Similarly, delivering RNA molecules to the target tissue or cells within the heart is a significant challenge^55^. RNA molecules, particularly unmodified RNAs, are susceptible to degradation by cellular nucleases and face barriers such as poor cellular uptake and limited intracellular trafficking^56^. Developing effective delivery systems, such as lipid nanoparticles, viral vectors, or other carriers, is essential to protect RNA molecules, enhance their stability, improve cellular uptake, and facilitate their release within the target cells. RNA-based therapeutics, such as ASOs and siRNAs, are designed to be highly specific to their target genes. However, unintended binding to off-target mRNA sequences can occur, leading to potential off-target effects and interfering with normal cellular processes^57^. Careful design and optimization of RNA sequences, as well as extensive characterization and validation, are necessary to minimize off-target effects and ensure the specificity of the therapeutic approach.

Furthermore, RNA molecules, especially when delivered exogenously, can trigger immune responses in the body^58^. This immune response can lead to the activation of the innate immune system, the release of pro-inflammatory cytokines, and potential adverse reactions^58^. The immunogenicity of RNA-based therapeutics needs to be carefully evaluated and mitigated to ensure their safety and minimize unwanted immune responses. Long-term safety considerations are crucial for RNA-based therapeutics, which may require prolonged treatment or repeated administration. Factors such as the potential for chronic inflammation, accumulation of RNA molecules, or interference with endogenous cellular processes need to be addressed through comprehensive preclinical and clinical studies. Monitoring for potential long-term side effects and ensuring the safety of patients receiving RNA-based therapies is of utmost importance.

It is also important to note that the manufacturing process for RNA-based therapeutics can be complex and costly, particularly for personalized or patient-specific treatments^59^. Developing scalable and cost-effective manufacturing methods is essential to enable broader accessibility and affordability of RNA-based therapeutics for HF and other diseases.

### Ongoing research efforts and technological advancements aimed at overcoming these challenges

Ongoing research efforts and technological advancements are focused on addressing the challenges and limitations associated with RNA-based therapeutics in HF.

Significant progress has been made in developing delivery systems for RNA-based therapeutics. Nanoparticle-based carriers, such as lipid nanoparticles, have shown promise in protecting RNA molecules from degradation, improving cellular uptake, and facilitating targeted delivery^60^. Advances in nanotechnology, such as the design of novel lipid formulations and surface modifications, are being explored to enhance delivery efficiency and tissue specificity.

Chemical modifications of RNA molecules are being investigated to improve their stability, cellular uptake, and immunogenicity profile. For example, incorporating modified nucleotides, such as 2ʹ-*O*-methyl or phosphorothioate linkages, in the RNA backbone can enhance stability and reduce immune activation^22^. Optimizing the type and position of chemical modifications is an active area of research to balance efficacy, specificity, and safety.

Advances in genomics, transcriptomics, and computational biology are aiding in identifying and validating potential target genes for RNA-based therapeutics. High-throughput screening approaches and bioinformatics tools identify key genes and signalling pathways involved in HF. This knowledge helps guide the design of RNA molecules specifically targeting the identified genes or gene products.

Strategies to minimize off-target effects are being explored to improve the specificity of RNA-based therapeutics. This includes refining the design of RNA sequences, optimizing chemical modifications, and utilizing bioinformatics tools to predict potential off-target interactions^61^. CRISPR/Cas9-based genome editing techniques are also being investigated to edit RNA sequences and mitigate off-target effects precisely.

Researchers are actively investigating methods to modulate the immunogenicity of RNA-based therapeutics. This includes designing less immunostimulatory RNA sequences, incorporating modified nucleotides, or co-delivering immunomodulatory agents to mitigate unwanted immune responses^62^. Understanding the mechanisms underlying the immune response to RNA molecules and developing strategies to modulate these responses is an area of active investigation.

Efforts are being made to develop scalable and cost-effective manufacturing processes for RNA-based therapeutics^63^. This includes optimization of RNA synthesis, purification methods, and formulation techniques to streamline the production process. Automation and robotics are employed to increase manufacturing efficiency and reduce costs, making RNA-based therapeutics more accessible.

### Potential future directions and implications for patients management

**Figure 2:**
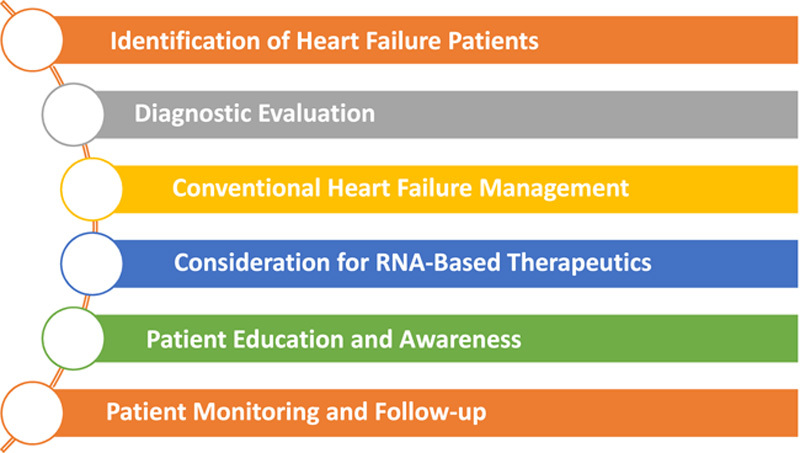
RNA-based therapeutics in the treatment algorithm for heart failure.

The future of RNA-based therapies for HF holds several exciting potential directions, including personalized therapies and combination approaches. See Figure 2.

The development of personalized RNA-based therapies involves tailoring treatments to individual patients based on their genetic profiles and disease characteristics^64^. With advancements in genomic sequencing technologies, it is becoming increasingly feasible to identify patient-specific genetic mutations or variations that contribute to HF. This information can be used to design RNA-based therapeutics that precisely target the identified genetic defects, allowing for more personalized and targeted interventions.

Combining different RNA-based therapies, such as ASOs, siRNAs, and mRNA therapies, with complementary mechanisms of action can have synergistic effects and enhance therapeutic outcomes. For example, combining an ASO targeting a specific gene with a siRNA targeting a different gene within the same signalling pathway may provide a more comprehensive and effective approach to modulating the disease. Integrating RNA-based therapeutics with small molecules or gene editing technologies, such as CRISPR/Cas9, holds promise for HF treatment. Small molecules can enhance the delivery and stability of RNA molecules or modulate specific cellular processes, amplifying the therapeutic effects. Gene editing technologies offer the potential to precisely correct disease-causing genetic mutations or manipulate gene expression, providing long-lasting therapeutic benefits.

In addition to targeting specific genes or signalling pathways, RNA-based therapeutics can promote cardiac regeneration and repair. mRNA-based therapies can be designed to induce the expression of factors that enhance tissue regeneration, such as growth factors or stem cell differentiation factors. By promoting the proliferation and differentiation of cardiac progenitor cells or enhancing the regenerative capacity of existing cardiomyocytes, RNA-based therapeutics can potentially restore damaged heart tissue and improve cardiac function.

Combining RNA-based therapies with existing treatment modalities for HF, such as pharmacological agents or medical devices, may offer synergistic effects and improved clinical outcomes. For example, combining RNA-based therapies with beta-blockers or angiotensin-converting enzyme (ACE) inhibitors, commonly used medications for HF, may have additive or synergistic effects on cardiac remodelling and function.

Continued advancements in delivery systems for RNA-based therapeutics will be crucial for their successful translation into clinical practice. Novel delivery methods, such as cell-specific targeting, tissue-specific targeting, or organ-specific targeting, can enhance the precision and efficacy of RNA-based therapies. Additionally, developing non-viral delivery systems, such as synthetic nanoparticles or exosomes, may offer improved safety profiles and scalability compared to viral vectors.

## Limitation of the study

One potential limitation of the review is the exclusion of non-English language articles. By focusing solely on English language publications, language bias is possible, which may result in excluding other relevant studies or findings published in other languages. However, the review provides a comprehensive overview of the potential of RNA-based therapeutics in revolutionizing HF management. It covers RNA-based therapies and their implications for personalized treatment, targeted therapy, combination approaches, and regenerative medicine. The breadth of coverage ensures a holistic understanding of the field.

## Conclusion

Over the years, HF has remained a significant burden on global healthcare systems, necessitating innovative approaches to improve patient outcomes. With their ability to target specific molecular pathways and modulate gene expression, RNA-based therapeutics offer a novel avenue for precision medicine in HF management.

The reviewed evidence demonstrates the potential of RNA-based therapeutics in addressing various aspects of HF, including myocardial remodelling, inflammation, fibrosis, and cardiac regeneration. By targeting specific genes and proteins involved in these pathological processes, RNA-based therapeutics can potentially halt or even reverse the progression of HF. Additionally, their versatility allows for personalized treatment strategies, considering individual patients’ unique genetic profiles.

The emergence of technologies such as ASOs, siRNAs, and mRNA-based vaccines has provided researchers with powerful tools to manipulate gene expression. These advancements have paved the way for innovative therapies targeting key molecular pathways involved in HF pathophysiology.

However, despite the promising potential, several challenges remain in translating RNA-based therapeutics from bench to bedside. Issues such as delivery methods, off-target effects, immunogenicity, and cost-effectiveness need to be addressed for widespread clinical application. Rigorous preclinical and clinical studies are crucial to establishing the safety and efficacy of these therapeutics, ensuring their regulatory approval and integration into standard clinical practice.

Nevertheless, the rapidly evolving field of RNA-based therapeutics holds great promise for transforming HF management. By targeting the disease’s underlying molecular mechanisms, these therapies can improve patient outcomes, reduce hospitalizations, and enhance the overall quality of life for individuals living with HF. Continued research, collaboration among scientists, clinicians, and pharmaceutical companies, and investment in infrastructure and resources are vital to unlocking the full potential of RNA-based therapeutics in the fight against HF.

## Ethical approval

Not applicable.

## Consent

Not applicable.

## Sources of funding

Not applicable.

## Author’s contribution

N.A.: conceptualization. All authors: writing of the first and the final draft.

## Conflicts of interest disclosure

The authors declare that there are no conflicts of interest.

## Research registration unique identifying number (UIN)

Not applicable.

## Guarantor

Nicholas Aderinto.

## Data availability statement

No new datasets were generated for this review.

## Provenance and peer review

Not commissioned, externally peer-reviewed.
